# A Comparative Study for the Incorporation of 8-oxo-dATP in DNA by Human DNA Polymerases

**DOI:** 10.3390/ijms27062537

**Published:** 2026-03-10

**Authors:** Alexander A. Kruchinin, Polina N. Kamzeeva, Mikhail S. Baranov, Yana G. Belova, Elizaveta O. Boldinova, Andrey G. Baranovskiy, Tahir H. Tahirov, Andrey V. Aralov, Alena V. Makarova

**Affiliations:** 1 Institute of Gene Biology, Russian Academy of Sciences, 34/5 Vavilova St., 119334 Moscow, Russia; 2 National Research Center “Kurchatov Institute”, Kurchatov sq. 1, 123182 Moscow, Russia; 3 Shemyakin-Ovchinnikov Institute of Bioorganic Chemistry, Russian Academy of Sciences, Miklukho-Maklaya 16/10, 117997 Moscow, Russia; 4 Eppley Institute for Research in Cancer and Allied Diseases, Fred & Pamela Buffett Cancer Center, University of Nebraska Medical Center, Omaha, NE 68198, USAttahirov@unmc.edu (T.H.T.)

**Keywords:** 8-oxo-dATP, DNA polymerase, mutagenesis, reactive oxygen species (ROS)

## Abstract

In this work, we analyzed the ability to incorporate 8-oxo-dATP by several human DNA polymerases: replicative Pol ε (exo-) from Family B; base excision repair (BER) enzymes Pol β and Pol λ from Family X; and translesion Pol η, Pol ι, and Pol κ from Family Y. We demonstrated that human DNA polymerases differ in their abilities to discriminate against 8-oxo-dATP. Among the tested DNA polymerases, Pol λ exhibited the worst ability to discriminate against 8-oxo-dATP opposite template T on DNA substrates with a protruding single-stranded 5′-end and a double-stranded DNA with a 1 nt gap. Pol β and DNA polymerases of Family Y showed relatively high accuracy. Pol η demonstrated the most effective discrimination against 8-oxo-dATP on templates T and G. Pol ι exclusively incorporated 8-oxo-dATP opposite template G but not T. Unexpectedly, the catalytic subunit of high-fidelity Pol ε (exo-) incorporated 8-oxo-dATP opposite templates T and G with higher efficiency compared with the error-prone polymerases of Family Y and Pol β. While the structures of human polymerases with incoming 8-oxo-dATP are not available, we speculate on a possible mechanism of 8-oxo-dATP discrimination.

## 1. Introduction

Guanine is the most susceptible DNA base to oxidation due to its low redox potential [1]. It readily forms a 7,8-dihydro-8-oxoguanine (8-oxoG) lesion when attacked by reactive oxygen species (ROS) in DNA [2,3]. Moreover, guanine in the nucleotide pool is also susceptible to oxidation, and its mutagenic potential can result from the incorporation of 2′-deoxy-7,8-dihydro-8-oxo-guanosine-5′-triphosphate (8-oxo-dGTP) into DNA [4,5]. The active site of some DNA polymerases can be less demanding with respect to the structure of DNA and/or incoming nucleotides, enabling the incorporation of modified nucleotides directly from the nucleotide pool. To enhance the selection of complementary canonical nucleotides during replication and DNA repair, the enzyme Human MutT Homolog 1 (MTH1) recognizes and hydrolyzes 8-oxo-dGTP to the corresponding monophosphate, preventing promutagenic events [6].

7,8-dihydro-8-oxoadenine (8-oxoA) is another abundant oxidative lesion with dual miscoding properties [7]. Compared with the parent adenine, the presence of the 8-oxo group in 8-oxoA increases the population of the *syn* conformer [8]. This conformer can form a stable Hoogsteen base pair with an opposing guanine, which leads to the A→C transversion mutations [9]. The number of 8-oxoA lesions ranges from 10 to 50% of 8-oxoG in DNA [10]. In some cells, it has been detected at the level of 0.7 lesions per 10^6^ nucleotides, which corresponds to approximately 2200 lesions per human genome and is comparable to 8-oxoG levels in mammalian cells [11]. 8-oxoA has been detected at elevated levels in cancer cells [12,13], and in some cases, the ratio of 8-oxoA to 8-oxoG reaches 1:1 [14].

The oxidation of adenine, like guanine, can also occur at comparable levels in the nucleotide pool, for example, after the treatment of HepG2 and LO2 cells with acetamiprid [15]. MTH1 exhibits catalytic activity towards 2′-deoxy-7,8-dihydro-8-oxoadenosine-5′-triphosphate (8-oxo-dATP) with efficiency comparable to that of 8-oxo-dGTP [16]. However, the incorporation of 8-oxo-dATP into DNA has not been studied comprehensively. Only a few DNA polymerases have been biochemically tested with 8-oxo-dATP [17]. In this work, we carried out a systematic analysis of the incorporation of 8-oxo-dATP into DNA by six human DNA polymerases belonging to several families. Our data demonstrate that human DNA polymerases vary in their abilities to discriminate against 8-oxo-dATP.

## 2. Results

In this work, we analyzed the ability of a number of human DNA polymerases involved in DNA translesion synthesis, DNA repair, and replication to incorporate 8-oxo-dATP into DNA. We tested the incorporation of dATP and 8-oxo-dATP in primer extension reactions opposite G and T at the +1 position of template DNA with a protruding single-stranded 5′-end. Additionally, the activity of base excision repair (BER) enzymes Pol β and Pol λ from Family X was analyzed on DNA templates containing a 1 nt gap.

### 2.1. Incorporation of 8-oxo-dATP by Translesion DNA Polymerases of Family Y

**Figure 1:**
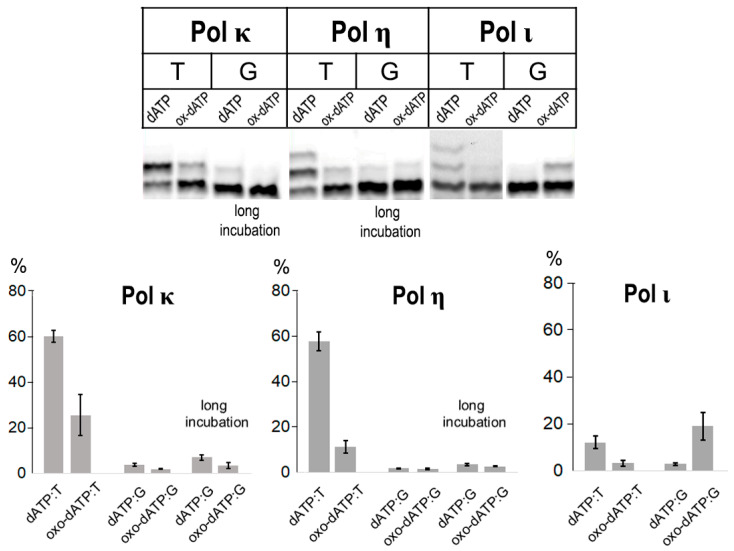
Incorporation of 8-oxo-dATP opposite templates T and G by human DNA polymerases of Family Y. Reaction conditions for Pol κ: standard 2 nM Pol and 2 min reaction time, long incubation 4 nM Pol and 4 min reaction time. Reaction conditions for Pol η: standard 5 nM Pol and 2 min reaction time, long incubation 10 nM Pol and 5 min reaction time. Reaction conditions for Pol ι: 15 nM Pol and 10 min reaction time. Mean values of primer extension and standard errors are indicated in diagrams.

Human DNA polymerases Pol η, Pol κ and Pol ι from Family Y play a key role as inserter polymerases during DNA translesion synthesis. Their ability to incorporate 8-oxo-dATP was analyzed for the first time. Pol η incorporated the control dATP opposite template T with high efficiency but successfully discriminated against the incorporation of 8-oxo-dATP regardless of the template nucleotide (Figure 1). Pol κ incorporated 8-oxodATP opposite template T more efficiently than Pol η. Pol κ and Pol η were inefficient on template G when incorporating non-complementary dATP and 8-oxo-dATP (even at higher enzyme concentrations and extended reaction times). Pol ι demonstrated low activity opposite template T. It is known that Pol ι prefers to incorporate dGTP over dATP and abrogates DNA synthesis opposite template T; this phenomenon has been called the “T-stop” rule [18]. Pol ι was unable to incorporate 8-oxo-dATP opposite template T, but moderate levels of 8-oxo-dATP incorporation by Pol ι were detected opposite template G (Figure 1).

### 2.2. Incorporation of 8-oxo-dATP by Pol β and Pol λ of Family X

**Figure 2:**
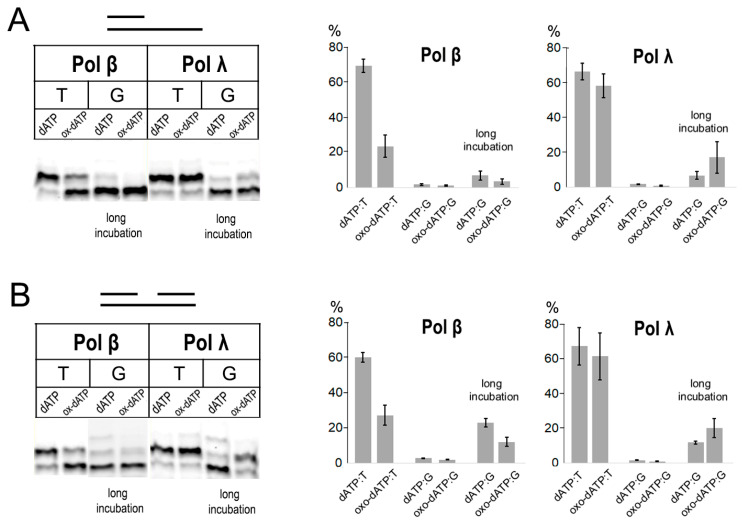
Incorporation of 8-oxo-dATP opposite templates T and G by human DNA polymerases of Family X. (**A**) Incorporation of 8-oxo-dATP by Pol β and Pol λ on DNA substrate with a protruding single-stranded 5′-end. Reaction conditions for Pol β: standard 4 nM Pol and 10 min reaction time, long incubation 60 nM Pol and 30 min reaction time. Reaction conditions for Pol λ: standard 4 nM Pol and 10 min reaction time, long incubation 60 nM Pol and 30 min reaction time. (**B**) Incorporation of 8-oxo-dATP by Pol β and Pol λ on DNA templates containing a 1 nt gap. Reaction conditions for Pol β: standard 2 nM Pol and 2 min reaction time, long incubation 60 nM Pol and 30 min reaction time. Reaction conditions for Pol λ: standard 5 nM Pol and 5 min reaction time, long incubation 60 nM Pol and 30 min reaction time. Mean values of primer extension and standard errors are indicated in diagrams.

Pol β and Pol λ are major DNA polymerases involved in DNA synthesis during BER. Their activity was tested on DNA with a protruding single-stranded 5′-end and double-stranded DNA substrates containing a 1 nt gap. Pol β possessed relatively high discriminatory behavior on both DNA substrates (Figure 2A,B). In particular, Pol β incorporated 8-oxo-dATP opposite template T 47-fold less efficiently than dATP on a DNA substrate with a protruding single-stranded 5′-end and 600-fold less efficiently on a double-stranded DNA substrate with a 1 nt gap (Table 1). Pol λ, in turn, demonstrated a low ability to discriminate the oxidized form of dATP. It was capable of incorporating both intact dATP and modified 8-oxo-dATP opposite template T on both DNA substrates with comparable efficiency (Figure 2A,B). Both Pol λ and Pol β incorporated nucleoside triphosphates opposite template G with low efficiency, but, unlike Pol β, Pol λ incorporated 8-oxo-dATP opposite G even better than canonical dATP (Figure 2A,B). We were unable to determine K_M_ for 8-oxo-dATP incorporation by Pol λ by steady-state kinetic analysis because K_M_ values were below working enzyme concentrations. Pol λ is known to have a significantly higher affinity for dNTPs compared with Pol β (37–130-fold lower K_M_) [19,20].

### 2.3. Incorporation of 8-oxo-dATP by Replicative B-Family Pol ε

**Figure 3:**
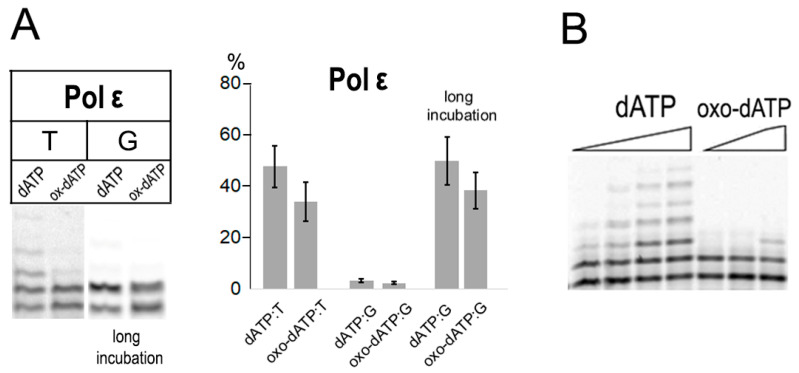
Incorporation of 8-oxo-dATP by human catalytic subunit Pol ε (exo-). (**A**) Incorporation of 8-oxo-dATP opposite templates T and G. Reaction conditions: standard 15 nM Pol and 5 min reaction time, long incubation 30 nM Pol and 30 min reaction time. Mean values of primer extension and standard errors are indicated in diagram. (**B**) Distributive character of 8-oxo-dATP opposite template T by Pol ε (exo-). Reactions were incubated for 10 min with 15 nM Pol ε for dATP and 20 min with 20 nM Pol ε for 8-oxo-dATP.

High-fidelity human Pol ε replicates the majority of the leading strand of genomic DNA [21]. Surprisingly, the Pol ε catalytic subunit variant lacking 3′-5′-exonuclease activity possessed a relatively low ability to discriminate against 8-oxo-dATP when incorporating opposite both templates T and G (especially under conditions of increased enzyme concentration and prolonged incubation time) (Figure 3A). Pol ε incorporated 8-oxo-dATP opposite T approximately 47-fold less efficiently than dATP, while both nucleotide substrates were incorporated with comparable efficiency opposite template G (Table 1). Unlike the incorporation of dATP opposite template T, Pol ε (exo-) was not able to continue replication beyond the non-canonical nucleotide substrate (Figure 3B).

## 3. Discussion

The concentrations of 8-oxo-dATP under physiological conditions and under oxidative stress can reach levels comparable with 8-oxo-dGTP [15]. However, the mutagenic effect of 8-oxo-dATP incorporation in DNA in mammalian cells has not been estimated yet. A single study indicates that 8-oxo-dATP can be effectively discriminated by DNA polymerases in vitro. Among a few tested enzymes, human Pol β demonstrated the most efficient incorporation of 8-oxo-dATP, especially opposite the complementary template T. Low incorporation efficiency was observed in reactions with *E. coli* KF and mammalian Pol α, which incorporated the oxidized nucleotide substrate preferentially opposite non-complementary G and A, while human Pol λ did not incorporate 8-oxo-dATP opposite any of tested DNA substrates [17]. Our data are in agreement with the relatively efficient incorporation of 8-oxo-dATP opposite template T by Pol β [17]. In contrast, our data indicate the very weak discrimination of 8-oxo-dATP opposite T and G on both single-stranded and gapped DNA substrates by human Pol λ.

Pol ε (exo-), Pol κ, Pol η and Pol ι were tested with 8-oxo-dATP in this work for the first time. Moderate (Pol κ, Pol ι) or limited (Pol η) incorporation of 8-oxo-dATP was observed by the translesion error-prone DNA polymerases of Family Y. Pol η demonstrated the most effective discrimination against 8-oxo-dATP (on templates T and G) among all tested enzymes, while Pol κ and Pol ι incorporated small amounts of 8-oxo-dATP opposite T and G, respectively. Remarkably, the catalytic subunit of high-fidelity Pol ε (exo-) showed weak ability to discriminate against 8-oxo-dATP opposite both template T and G nucleotides.

**Figure 4:**
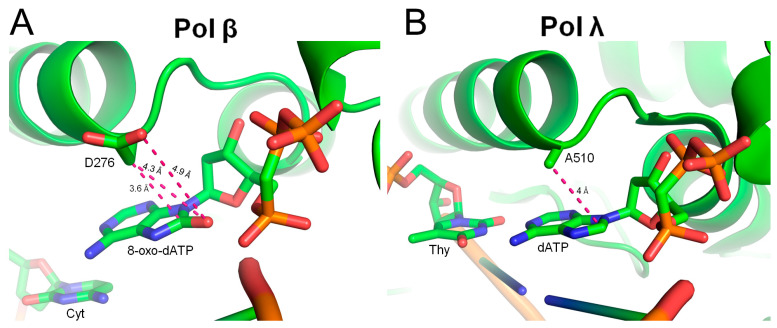
The active sites of the DNA polymerases of Family X. (**A**) Pol β with incoming 8-oxo-dATP (modeled from [22], PDB ID 3C2L). The distances between Asp276 and the 8-oxo group are indicated. (**B**) Pol λ with incoming dATP (from [28], PDB ID 4FO6). The distances are depicted by pink dashed lines.

The ability to discriminate against 8-oxo-dATP during DNA synthesis depends on the unique architectures of polymerases’ active sites. Human DNA polymerase structures with incoming 8-oxo-dATP are not yet available. We speculate that 8-oxo-dATP incorporation opposite template T occurs in the *anti*-conformation and may cause steric hindrance between the 8-oxo group and the triphosphate group or Me^2+^ ions in Pol β [22,23] (Figure 4A), Pol η [24], Pol ι [25] and Pol kappa [26] (Figure S1). This hindrance could result in a shift in the base and triphosphate of the incoming nucleotide (phosphate backbone of templating nucleotide might also be affected) and less efficient pairing with T (Figure S1). Moreover, residues Asp276 in Pol β (Figure 4A) and Tyr112 and Tyr138 in Pol κ (Figure S1) might clash with the rotating oxidized base, thereby contributing to 8-oxo-dATP discrimination [22,24,26]. In Pol ε, the 8-oxo group may cause steric hindrance with the deoxyribose ring, possibly affecting the sugar and base conformation [27] (Figure S2). Therefore, the efficiency of 8-oxo-dATP incorporation by a polymerase likely depends on the ability of its active site to rotate the base to accommodate the modification.

Remarkably, Pol λ was the most efficient polymerase for incorporating 8-oxo-dATP opposite template T. Indeed, it seems that only the Pol λ active site lacks serious steric hindrance with the 8-oxo-group of incoming 8-oxo-dATP. Unlike Pol β, which contains Asp276 close to the incoming dATP opposite a templating T (Figure 4A), the Pol λ active site at a similar position contains Ala510, which interferes less with the accommodation of the 8-oxo group [28] (Figure 4B) and allows for 8-oxo-dATP incorporation at higher extent. In the Pol β active site, Asp276 restricts the rotation of adenine (the steric hindrance between the C8 atom of Ade and Cβ atom of Asp276) when it tries to avoid a clash with the triphosphate group (Figure 4A). 

Pol ι, Pol λ and Pol ε (exo-) also demonstrated minor incorporation of 8-oxo-dATP opposite template G. Such activity likely requires the rotation of the oxidized adenine into the *syn* conformation and formation of Hoogsteen hydrogen bonds with the templating G. A similar accommodation of oxidized dGTP was demonstrated for incoming 8-oxo-dGTP opposite template A in the active sites of Pol λ and Pol β [22,28]. On the other hand, templating G can adopt a *syn* conformation. The formation of Hoogsteen base pairs opposite purines has been described for Pol ι [29]. Either way, the bulky G-Ade mismatch should stretch the active site and facilitate the accommodation of the oxidized incoming Ade.

## 4. Materials and Methods

### 4.1. Equipment and Reagents for Chemical Synthesis

All reagents were purchased from Sigma-Aldrich (Saint Louis, MO, USA). Solvents were purchased from CHIMMED (Moscow, Russia). ^1^H and ^31^P NMR spectra were recorded on a Bruker Avance III 600 spectrometer (Karlsruhe, Germany) at 600 and 243 MHz, respectively. The multiplicity of signals in the spectra is shown using the following abbreviations: s (singlet), d (doublet), t (triplet), quartet (q) and m (multiplet). The spin–spin coupling constants (J) are given in Hz. Ion-exchange chromatography was performed on an Akta Explorer 100 instrument (Cytiva, Uppsala, Sweden). The ESI HR mass spectrum was acquired on an LTQ FT Ultra (Bremen, Germany) mass spectrometer in a positive ion mode.

### 4.2. 8-oxo-dATP Synthesis

**Figure 5:**
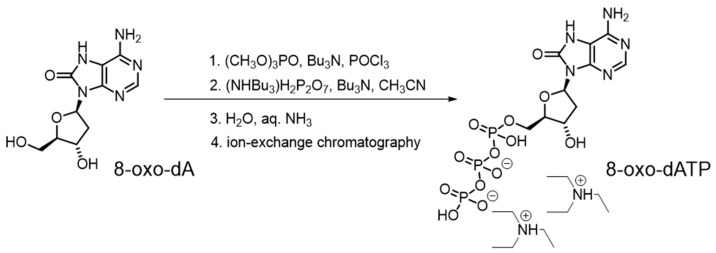
The synthesis of the bis-triethylammonium salt of 8-oxo-dATP.

An 8-oxo-dATP substrate was prepared from 2′-deoxy-7,8-dihydro-8-oxo-adenosine (8-oxo-dA) as was previously described for the synthesis of 8-oxo-εATP [30] with minor modification (Figure 5). Tributylamine (Bu_3_N, 1.43 mL) and freshly distilled trimethyl phosphate ((CH_3_O)_3_PO, 12.0 mL) were added to 8-oxo-dA [31] (0.80 g, 3.00 mmol) in a Schlenk flask (100 mL) under inert gas atmosphere. The mixture was vigorously stirred at room temperature for 30 min and then cooled to −10 °C. Under inert gas atmosphere, phosphorus oxychloride (POCl_3_, 0.50 mL, 5.40 mmol) was added to the reaction mixture, and the mixture was stirred at −10 °C for 1 h. Then, a phosphorylating agent prepared by vigorously stirring acetonitrile (CH_3_CN, 30 mL), Bu_3_N (4.2 mL, 17.7 mmol) and bis(tributylammonium)pyrophosphate ((NHBu_3_)_2_H_2_P_2_O_7_, 1.8 g, 3.30 mmol) under inert atmosphere for 20 min at −20 °C was added to the reaction mixture dropwise. After stirring the mixture at −10 °C for 1 h, cold water (65 mL) was added. The mixture was stirred at 0 °C for 1 h, then transferred to a separatory funnel, and washed with methylene chloride (25 mL, 5 times). The aqueous layer was collected, and aqueous ammonia solution was added to adjust the pH to 7.0. The resulting 8-oxo-dATP solution was stored in a refrigerator until purification by ion-exchange chromatography on a 50 × 250 mm column packed with HEMA-BIO 1000 DEAE 70 μm sorbent (Nürnberg, Germany) with a gradient of 50–600 mM triethylammonium bicarbonate (pH 7.6). Fractions containing the target product were evaporated. The residue was re-dissolved in water and evaporated to remove residual buffer to afford the bis-triethylammonium salt of 8-oxo-dATP (0.86 g, 1.21 mmol, 40%) as a white flaky powder. ^1^H NMR (600 MHz, D_2_O): δ 8.21 (s, 1H), 6.37 (d, J = 7.1 Hz, 1H), 4.83–4.78 (m, 1H), 4.32–4.27 (m, 1H), 4.26–4.22 (m, 1H), 4.20–4.15 (m, 1H), 3.34–3.29 (m, 1H), 3.25 (q, J = 7.3 Hz, 12H), 2.41–2.36 (m, 1H), 1.33 (t, J = 7.3 Hz, 18H). ^31^P NMR (243 MHz, D_2_O): δ -10.9 (d, J = 19.8 Hz, 1P), -11.2 (d, J = 20.0 Hz, 1P), -23.3 (dd, J = 19.8 Hz, J = 20.0 Hz, 1P). HRMS (ESI) m/z: calculated for C_10_H_17_N_5_O_13_P_3_^+^ [M - 2(C_2_H_5_)_3_N + H]^+^: 508.0030; found 508.0037. The ^1^H and ^31^P NMR spectra are shown in Figure S3 and Figure S4 of the Supplementary Materials, respectively.

### 4.3. DNA Templates and Enzymes

Pol η, Pol ι, Pol β, and Pol λ were purified from *S. cerevisiae* and *E. coli* as described in [32,33,34]. Human Pol κ was kindly provided by L.V. Gening (Institute of Molecular Genetics, Moscow, Russia). To express the catalytic core of the catalytic subunit of human Pol ε (a.a. 27–1172) lacking 3′-5′-exonuclease activity, we used plasmid pASHSUL-His6-Sumo-p261(27–1172)Δ(185–209)exo- [35]. The *POLE* gene was fused with the N-terminal 6xHIS-SUMO tag, the distorted region 185–209 a.a. was deleted and D275N/E277Q substitutions were introduced by site-directed mutagenesis. Protein expression was carried out in the C3013 *E. coli* strain at 15 °C for 12 h following induction with 0.15 µg/mL tetracycline. After metal affinity chromatography with Ni^2+^-NTA agarose, the SUMO tag was removed by Ulp protease, and the enzyme was purified on a heparin–Sepharose column.

The DNA oligonucleotides used in this study (Table 2) were synthetized as described previously [36]. To prepare DNA substrates, the 5′-Cy5-labeled primer Cy5-Pr16 was annealed to the corresponding unlabeled template oligonucleotides at a molar ratio of 1:1.1 in 100 mM NaCl by heating to 97 °C and slowly cooling to 4 °C.

### 4.4. DNA Polymerase Reactions for the Primer Extension Assay

Standard primer extension reactions were performed in 20 µL containing 100 nM DNA substrate, 50 μM dNTP (including 8-oxo-dATP), 30 mM HEPES pH 7.4, 10 mM MgCl_2_ (for Pol ε, Pol κ, Pol η, Pol β) or 1 mM MgCl_2_ (for Pol ι and Pol λ), 100 µg/mL BSA, 1 mM DTT, 8% glycerol and 2–60 nM of polymerase, as indicated in the figure legends. Reactions were incubated at 37 °C for 2–60 min, as indicated in figure legends, and placed on ice. Reactions were stopped by the addition of an equal volume of 2x loading buffer (20 mM EDTA, 0.001% bromophenol blue, 96% formamide) and heated for 5 min at 95 °C. The reaction products were resolved on 21% polyacrylamide gels with 8 M urea, visualized on Typhoon 9400 (GE Healthcare, Chicago, IL, USA) and analyzed with ImageQuant v8.2 software. The percent of the extended primer (PrExt) was calculated for each reaction, and the mean values of PrExt with the standard errors are shown in figures. The experiments were repeated three times.

### 4.5. Steady-State Kinetic Analysis of dNMP Incorporation

To quantify the incorporation of individual dNMPs, dNTP concentrations were varied from 0.1 to 2500 µM. The reactions were incubated at 37 °C with 0.35–80 nM Pol β and 10–20 nM Pol ε for different times to ensure that less than 30–40% of the primer is utilized at the highest dNTP concentration. The Michaelis constant (*K*_M_) and rate constant (*k*_cat_) were calculated by fitting the rate data to the Michaelis–Menten equation. Calculations were done using GraphPad Prism software 8.0.1 (Erithacus Software, East Grinstead, UK). The catalytic efficiency of dNMP incorporation was calculated as *k*_cat_/*K*_M_. The experiments were repeated 2–4 times.

## 5. Conclusions

Our data show that human DNA polymerases significantly differ in their ability to discriminate against 8-oxo-dATP during DNA synthesis. While some enzymes, such as Pol λ and Pol ε, may contribute to 8-oxo-dATP-induced mutagenesis, the exact deleterious effect of dATP oxidation in human cells is still unclear and will be a subject of future studies.

## Figures and Tables

**Table 1:** Steady-state kinetics analysis of dATP and 8-oxo-dATP incorporation by Pol β and Pol ε.

dATP/8-oxo-dATP:Template	Enzyme	DNA	K_M_, µM	k_cat_, min^−1^	k_cat_/K_M_ (min^−1^ μM^−1^)
dATP:T	Pol β	1 nt gap	15.2 ± 2.4	54.6 ± 4.9	3.78 ± 0.65
		5′-overhang	95.3 ± 25.3	19.5 ± 4.3	0.24 ± 0.06
	Pol ε	5′-overhang	1.9 ± 0.3	1.1 ± 0.3	0.61 ± 0.20
8-oxo-dATP:T	Pol β	1 nt gap	54.5 ± 11.6	0.33 ± 0.05	0.0063 ± 0.0007
		5′-overhang	22.1 ± 12.8	0.09 ± 0.02	0.0051 ± 0.0020
	Pol ε	5′-overhang	14.4 ± 8.4	0.16 ± 0.06	0.013 ± 0.003
dATP:G	Pol β	1 nt gap	181.6 ± 25.5	0.013 ± 0.002	0.00008 ± 0.00001
		5′-overhang	140.3 ± 90.4	0.0024 ± 0.0004	0.000026 ± 0.000001
	Pol ε	5′-overhang	108.3 ± 25.9	0.17 ± 0.03	0.0016 ± 0.0001
8-oxo-dATP:G	Pol β	1 nt gap	144.2 ± 18.0	0.012 ± 0.001	0.00009 ± 0.00002
		5′-overhang	89.3 ± 31.4	0.0009 ± 0.0001	0.000014 ± 0.000006
	Pol ε	5′-overhang	96.3 ± 18.8	0.15 ± 0.03	0.002 ± 0.001

**Table 2:** Oligonucleotides used in this study.

Oligonucleotide	Sequence 5′-3′
Cy5-Pr16	Cy5-GTCACAGAGATACTAC
TemplateTA	GAGCAGTCGCACATGTAGTATCTCTGTGAC
TemplateGA	GAGCAGTCGCACAGGTAGTATCTCTGTGAC
Closing_8-oxo-dATP	/P/-TGTGCGACTGCTC

P = 5′-phosphate. The +1 positions of templating T and G nucleotides are underlined.

## Data Availability

The original contributions presented in this study are included in the article/Supplementary Material. Further inquiries can be directed to the corresponding authors.

## Supplementary Materials

The following supporting information can be downloaded at https://www.mdpi.com/article/10.3390/ijms27062537/s1.
