# Supplementary material for: A Comparative Study for the Incorporation of 8-oxo-dATP in DNA by Human DNA Polymerases
Source: Int J Mol Sci. 2026 Mar 10;27(6):2537. doi: 10.3390/ijms27062537 (PMC13026661; doi:10.3390/ijms27062537)
Supplement: Supplementary file 1 [file ijms-27-02537-s001.zip › ijms-4070488-supplementary.pdf]

Supplementary Material

A comparative study for incorporation of 8-oxo-dATP into DNA by human DNA polymerases

Alexander A. Kruchinin [1,2], Polina N. Kamzeeva [1,3], Mikhail S. Baranov [3], Yana G. Belova[2], Elizaveta O. Boldinova[1,2], Andrey G. Baranovskiy[4], Tahir H. Tahirov[4], Andrey V. Aralov [3,,5*] and Alena V. Makarova [1,2,*]

1 Institute of Gene Biology, Russian Academy of Sciences, 34/5 Vavilova St., 119334 Moscow, Russia; kruchinin77@gmail.com (A.A.K.); lizaboldinova@yandex.ru (E.O.B.)
2 National Research Center "Kurchatov Institute", Kurchatov sq. 1, 123182 Moscow, Russia yana_k6798@mail.ru
3 Shemyakin-Ovchinnikov Institute of Bioorganic Chemistry, Russian Academy of Sciences, Miklukho-Maklaya 16/10, 117997 Moscow, Russia; polinabast@yandex.ru (P.N.K.); baranovmikes@gmail.com (M.S.B)
4 Eppley Institute for Research in Cancer and Allied Diseases, Fred & Pamela Buffett Cancer Center, University of Nebraska Medical Center, Omaha, NE 68198, USA; abaranovskiy@unmc.edu, ttahirov@unmc.edu
5 Educational Resource Center for Cellular Technologies, RUDN University, 117198 Moscow, Russia
* Correspondence: baruh238@mail.ru (A.V.A.); amakarova-img@yandex.ru (A.V.M.)

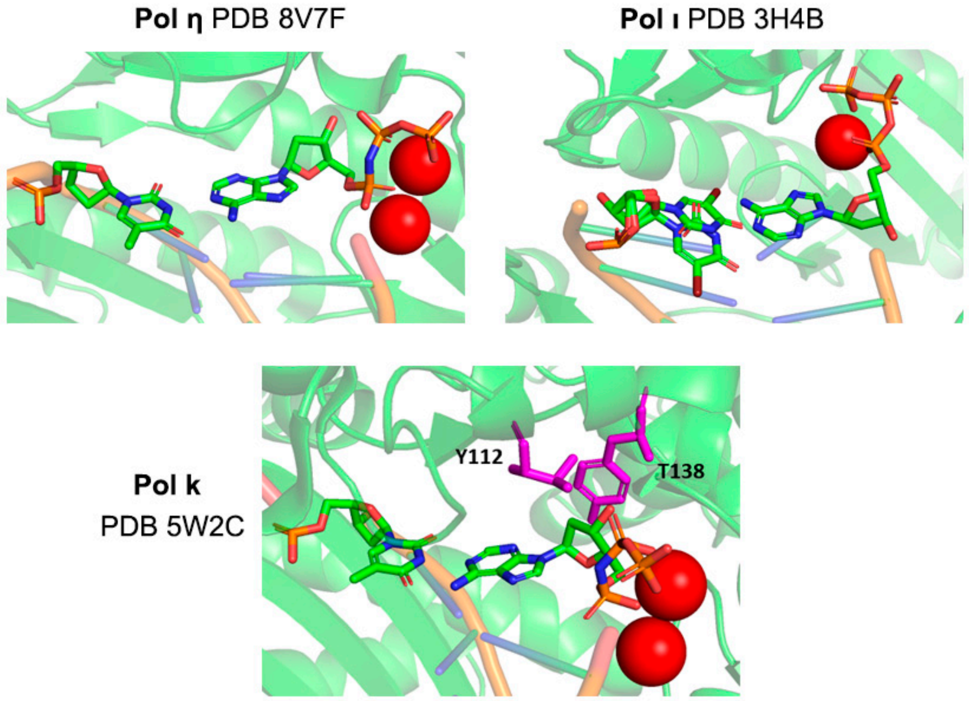

Figure S1. Structures of human DNA polymerases of Family Y. Pol η – incoming dAMPNPP opposite T, PDB 8V7F [https://doi.org/10.2210/pdb8V7F/pdb]. Pol ι – incoming dATP opposite T and dU, PDB 3H4B [https://doi.org/10.2210/pdb3H4B/pdb]. Pol κ – incoming dAMPNPP opposite T on DNA with Lucidin-derived DNA adduct, PDB 5W2C [https://doi.org/10.2210/pdb5W2C/pdb].

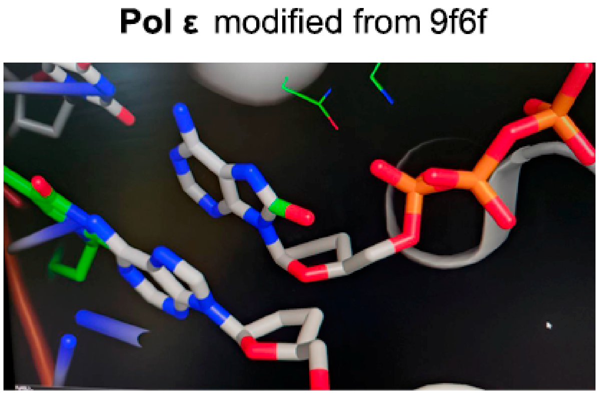

Figure S2. Structure of human Pol ε with incoming 8-oxo-dATP opposite template T. The 8-oxo group in Pol ε was added from 9F6F [https://doi.org/10.2210/pdb9F6F/pdb] using the Pymol editing tool.

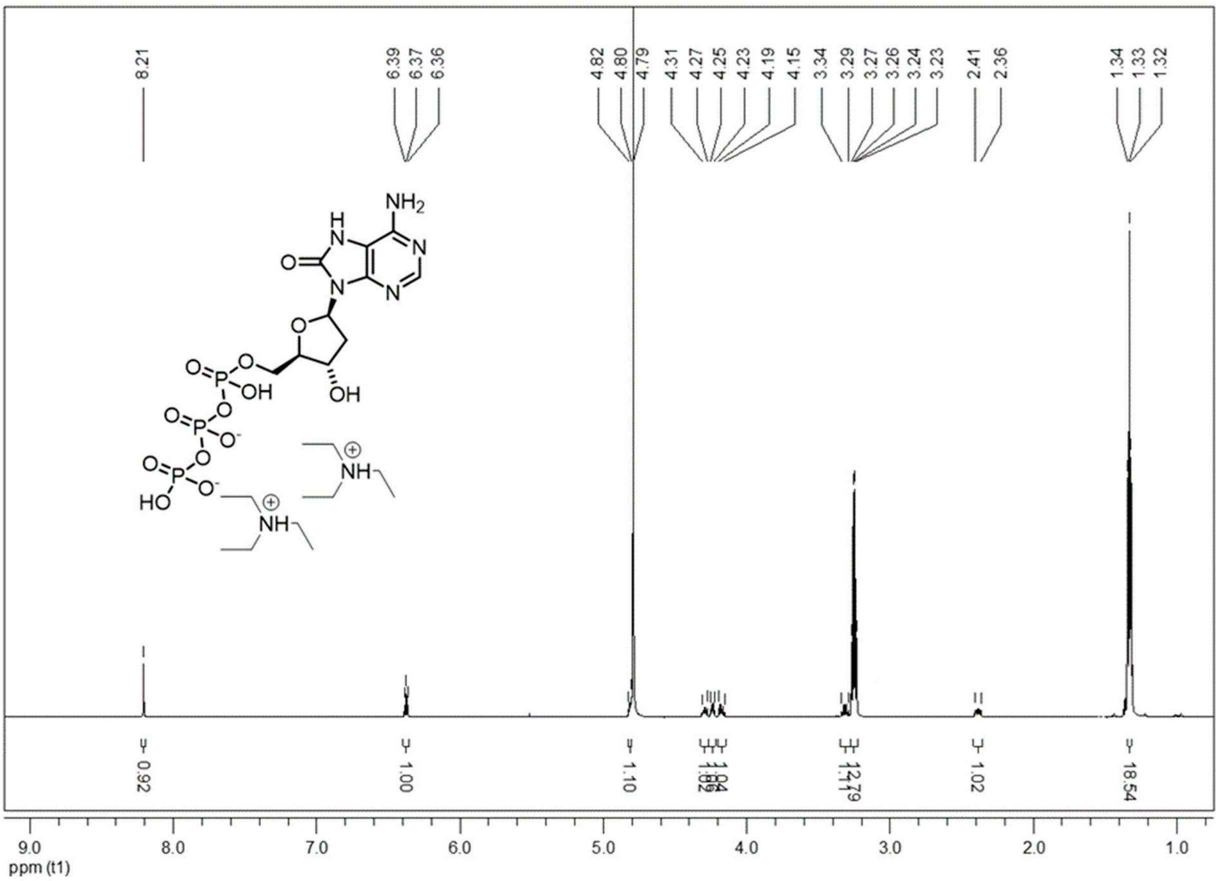

Figure S3. ¹H NMR spectrum of the bis-triethylammonium salt of 8-oxo-dATP

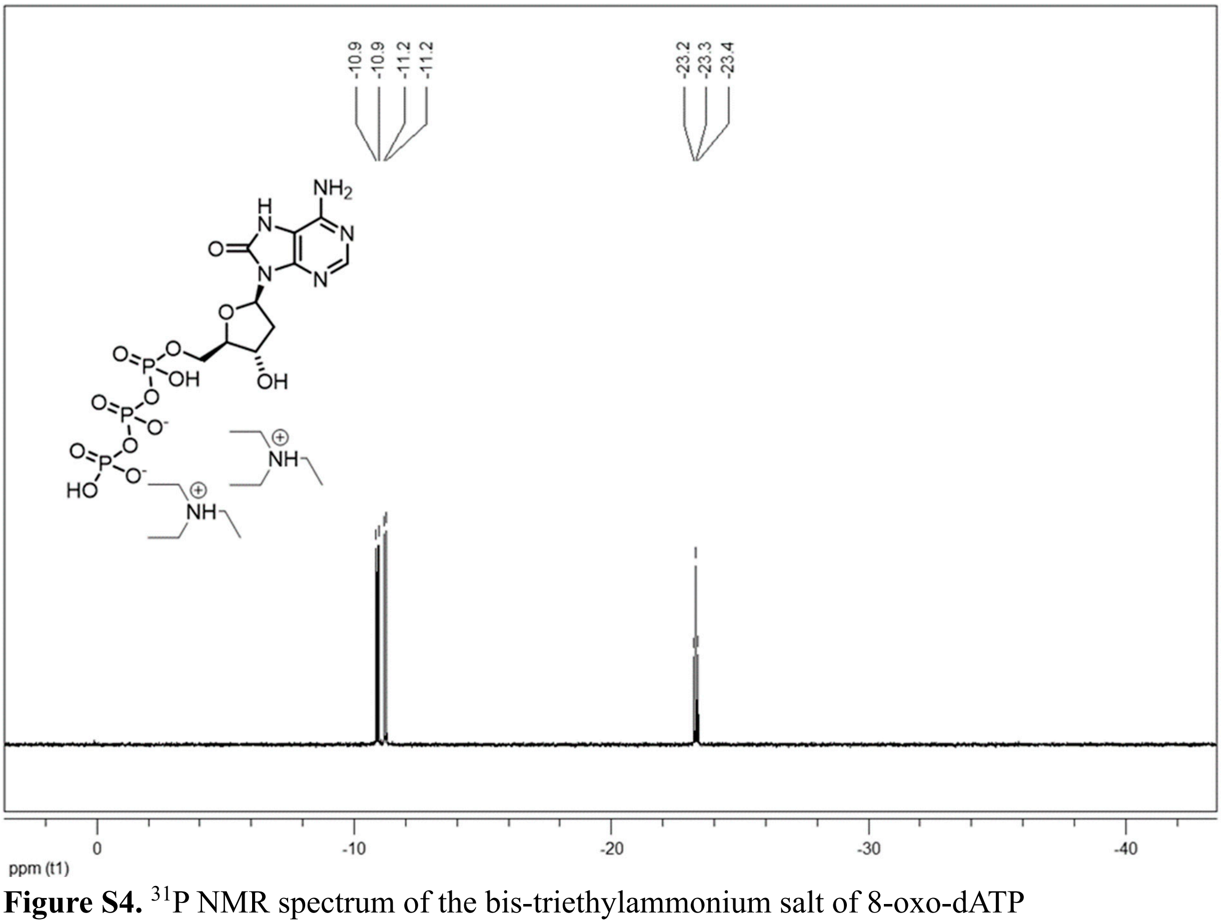

Figure S4. ³¹P NMR spectrum of the bis-triethylammonium salt of 8-oxo-dATP
